# Asymptomatic Meckel’s Diverticulum in Adults: Is Diverticulectomy Indicated?

**DOI:** 10.4103/1319-3767.65199

**Published:** 2010-07

**Authors:** Leo F. Tauro, Celine George, Bangalore S. Rao, John J. Martis, Leo T. Menezes, Hejmadi D. Shenoy

**Affiliations:** Department of General Surgery, Fr. Muller Medical College Hospital, Kankanady, Mangalore (D.K.), Karnataka, India

**Keywords:** Asymptomatic, gut duplication, Meckel’s diverticulum, surgery

## Abstract

**Background/Aim:**

The objective of this study was to estimate the incidence of the Meckel’s diverticulum (MD) and to study its clinical profile and surgical outcome, as well as to check whether diverticulectomy is indicated for asymptomatic MD in adults.

**Materials and Methods:**

This is a prospective study of 1332 patients who were operated upon for acute abdomen during the period August 1999 to July 2009 in a single surgical unit. Preoperative abdominal ultrasonography and plain x-ray abdomen (erect) were done depending on the necessity. These patients were subjected to laparotomy/ appendicectomy depending on the case. A search for MD was done, and if found, surgical resection and analysis by histopathological confirmation of the resected MD were performed.

**Results:**

During the operation, this study detected 15 (1.13%) patients with MD. In none of these cases, preoperative diagnosis of Meckel’s diverticulitis was made. The age of the patients ranged from 18 to 68 years (mean age, 32.9 years). Out of 15 patients, 9 (60%) were males; 6 (40%) were females. Seven (46.7%) cases were symptomatic due to MD and 8 (53.3%) were asymptomatic. One patient presented with hematochezia; 2, with intestinal obstruction due to gangrene of the MD; and 4, with Meckel’s diverticulitis. One patient had duplication of (double) Meckel’s diverticulum without any inflammation in both the diverticulae. Histopathological examination of these specimens confirmed 4 cases with inflammation; 2, with gangrene; and 1, with ulcerated gastric mucosa in the MD. Among these, in 2 (13.3%) cases there was heterotopic epithelium (ulcerated gastric mucosa- 1, colonic mucosa- 1).

**Conclusion:**

We recommend that a search for MD in every case of appendicectomy/ laparotomy done for acute abdomen should be conducted, and if found, Meckel’s diverticulectomy or resection should be performed to avoid secondary complications arising from it.

Meckel’s diverticulum (MD) is present in 2% (0.3%-2.5%) of the population; it is situated on the antimesenteric border of the small intestine, commonly 60 cm from the ileocecal valve, and is usually 3 to 5 cm long (2% incidence - 2 feet from the ileocecal valve – 2 inches long). There is a male preponderance, with male-to-female ratio being approximately 3:2. Preoperative diagnosis of MD is very difficult.[1,2] MD is very often fortuitously discovered; and if its resection is not done immediately because of local conditions, fatal complications can occur.[2,3]

The present study was conducted to (1) estimate the incidence of MD, (2) study its clinical profile and surgical outcome and (3) check whether diverticulectomy is indicated for asymptomatic MD in adults.

## MATERIALS AND METHODS

This is a prospective study of 1332 patients who were operated upon for acute abdomen during the period August 1999 to July 2009 (10 years) in a single surgical unit. Preoperative abdominal ultrasonography (USG) and plain x-ray abdomen (erect) were done depending on the necessity. Abdominal USG was done in all patients, but plain x-ray abdomen (erect) was done in 5 patients [Table 1], out of whom 1 patient (22-year-old male) was suspected to have perforated appendicitis. All these patients were subjected to laparotomy/ appendicectomy depending on the diagnosis. The incisions used were Lanz, Rutherford-Morrison’s, midline and right lower para-median incisions [Table 2]. Midline and para-median incisions were extended as per need. A search for MD was done, and if found, diverticulectomy or resection was performed. Diverticulectomy (cuneiform resection) was performed in 6 patients who had narrow-based MD, and resection of MD with adjacent segment of ileum on either side was performed in the remaining 9 patients who had broad-based MD. Thorough peritoneal toileting wherever necessary was done, and a proper wound toileting was done in every case after peritoneal closure. These patients were followed up postoperatively. Histopathological examination of resected MD and the study of diverticular mucosal epithelial pattern were done.

**Table 1:** Details of patients who had plain X-ray abdomen (erect)

Preoperative diagnosis	No. of patients
Acute intestinal obstruction	3
Acute peritonitis	1
Acute appendicitis	1
Total	5

**Table 2:** Types of incisions used for appendicectomy/laparotomy

Type of incision	No. of patients
Lanz	8
Rutherford-Morrison’s	1
Upper midline	2
Lower midline	1
Right para-median	3
Total	15

## RESULTS

**Figure 1:**
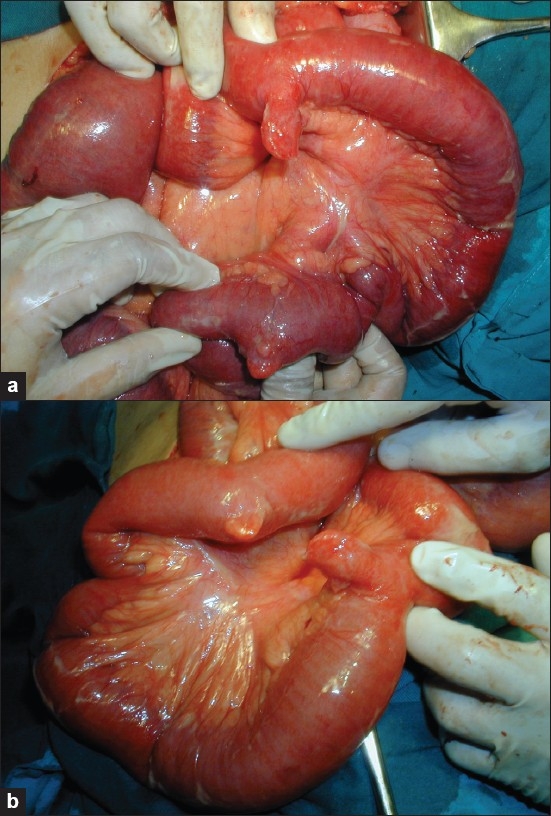
(a and b) Photograph showing double Meckel’s diverticulum

During the operation, this study detected 15 (1.13%) patients with MD. In none of these patients, presence of MD was detected preoperatively. Out of 15 patients, 9 (60%) were males and 6 (40%) were females. Age of the patients ranged from 18 to 68 years (mean age, 32.9 years). Seven (46.7%) patients were symptomatic due to MD, and the remaining 8 (53.3%) were asymptomatic. One patient presented with hematochezia; 2, with intestinal obstruction due to gangrene of the Meckel’s diverticulum; and 4, with Meckel’s diverticulitis. One patient had duplication of (double) Meckel’s diverticulum without any inflammation in both the diverticulae [Figures 1a and 1b]. Histopathological examination of these specimens confirmed 4 cases with inflammation; 2, with gangrene; and 1, with ulcerated gastric mucosa in the MD [Table 3]. Among these, in 2 (13.3%) cases there was heterotopic epithelium (ulcerated gastric mucosa- 1, colonic mucosa- 1). All these patients recovered without any major postoperative complication [Table 4].

**Table 3:** Profile of Meckel’s diverticulum in our study

Age (Yrs.)	Sex	Preoperative diagnosis	Postoperative diagnosis	Histopathology of MD	Mucosal epithelium of MD
21	F	Acute appendicitis	Acute appendicitis	Meckel’s with no inflammation	Intestinal
18	F	Acute appendicitis	Meckel’s diverticulitis	Inflamed Meckel’s	Intestinal
30	M	Acute appendicitis	Acute appendicitis	Meckel’s with no inflammation	Colonic
68	M	Hematochezia	Ulcerated Meckel’s diverticulum	Meckel’s with ulcerated mucosa	Gastric
60	M	Acute intestinal obstruction	Acute intestinal obstruction due to adhesions	Meckel’s with no inflammation	Intestinal
48	M	Acute intestinal obstruction	Acute intestinal obstruction due to gangrenous Meckel’s diverticulum	Meckel’s with gangrenous changes	Intestinal
21	F	Acute appendicitis	Meckel’s diverticulitis	Inflamed Meckel’s	Intestinal
18	M	Acute appendicitis	Acute appendicitis	Meckel’s with no inflammation	Intestinal
40	M	Acute peritonitis	Acute peritonitis due to perforated appendix	Meckel’s with no inflammation (double Meckel’s)	Intestinal in both
25	F	Acute appendicitis	Acute appendicitis	Meckel’s with no inflammation	Intestinal
40	M	Acute appendicitis	Acute appendicitis	Meckel’s with no inflammation	Intestinal
28	F	Acute appendicitis	Meckel’s diverticulitis	Inflamed Meckel’s	Intestinal
20	M	Acute appendicitis	Acute appendicitis	Meckel’s with no inflammation	Intestinal
22	M	Acute intestinal obstruction	Acute intestinal obstruction due to gangrenous Meckel’s diverticulum	Meckel’s with gangrenous changes	Intestinal
35	F	Acute appendicitis	Meckel’s diverticulitis	Inflamed Meckel’s	Intestinal

**Table 4:** Morbidity and mortality of Meckel’s diverticulum resection

Clinical presentation	Complications	No. of patients	Morbidity (%)	Mortality (%)
Symptomatic	Wound collection/infection	2	28.6	Nil
Asymptomatic	Subacute intestinal obstruction	1	12.5	Nil

## DISCUSSION

MD represents the patent intestinal end of the vitellointestinal duct. It possesses all three coats of the intestinal wall and has its own blood supply. It is therefore vulnerable to infection and obstruction in the same way as the appendix. In 20% of the cases, the mucosa contains heterotopic epithelium, namely, gastric, colonic or sometimes pancreatic tissue.[1,2]

The symptoms of MD are 1) severe hemorrhage caused by peptic ulceration (the blood is passed per rectum); 2) intussusception; 3) Meckel’s diverticulitis, with or without perforation; 4) chronic peptic ulceration; 5) intestinal obstruction due to the presence of a band between the apex of the diverticulum and the umbilicus. MD is very difficult to demonstrate by contrast radiology; small bowel enema would be the most accurate investigation. Technetium (TC)- 99m scanning may localize heterotopic gastric mucosa, revealing the site of an MD in 90% of cases. Treatment is Meckel’s diverticulectomy or intestinal resection,[1,2] i.e., cuneiform resection of MD or resection of MD with an adjacent segment of ileum on both sides.

Cennamo *et al*.,[3] who studied for the presence of MD in 1211 patients affected by appendiceal-like pathology, recommend that the diverticulum should be always totally removed even when it is asymptomatic, because the problems arising as secondary complications are more severe compared to any discomfort following its surgical removal. Aarnio *et al*.[4] studied 71 patients with MD and recommended that it should be searched in the laparotomy due to acute abdomen. In his retrospective analysis, 46 (65.5%) males and 25 (34.5%) females. MD was found during 55 (1.5%) out of 3758 appendicectomies.[4] The age of the patients ranged from 11 months to 87 years (mean, 30.4 years). Preoperatively, the diagnosis was made in 3 patients - two patients with TC-99m scanning and 1 patient with intestinal passage radiography. Forty-six Meckel’s diverticulae were asymptomatic, but 25 (34.5%) cases were symptomatic. Nine patients had ulcer in the diverticulum, which was perforated in 5 cases. Eight patients had intestinal occlusion, 5 patients had Meckel’s diverticulitis, 2 patients had invagination and in 1 case a sharp piece of plastic material had perforated the MD.

Albu *et al*.[5] reported a case of double (duplication) Meckel’s diverticulum. We[6] encountered another case of double Meckel’s diverticulum. Duplications of the alimentary tract are rare congenital malformations. The small bowel is the commonest site of alimentary tract duplication. The patients may present with abdominal mass, distension, pain, vomiting, melena, perforation or obstruction.[7,8] We encountered a similar case in our study. There was a case report by Janusz[9] on mechanical occlusion of alimentary tract caused by the adhesion of gangrene-related changes in the MD. Prall *et al*.[10] reported another case of intestinal obstruction due to MD. The present study also includes 2 similar cases. Wilhelm *et al*.[11] reported 1 case of Meckel’s diverticulitis diagnosed by ultrasound. Zulfikaroglu *et al*.[12] studied asymptomatic MD and concluded that resection of incidentally found MD is not associated with increased operative morbidity and mortality. Karaman *et al*.[13] advocated prophylactic diverticulectomy in asymptomatic MD.

MD is difficult to diagnose preoperatively. Technitium-99m scanning and small bowel enema (enteroclysis) would be the most accurate investigations. Intestinal passage radiography includes barium meal follow-through and enteroclysis. In barium meal follow-through, the patient is given 16 ounces of barium orally and radiographs are taken at regular intervals until the barium column reaches the cecum. Compression spot radiographs of suspicious areas are taken. Water-soluble iodinated contrast can be used in cases of suspected perforations instead of barium. In enteroclysis, the patient is orally or nasally intubated and a small bowel tube is positioned with its tip beyond the ligament of Treitz. High-density barium is injected into the tube (200-250 cc), followed by injection of methyl cellulose or water as the double-contrast agent. The barium coats the mucosa and the methyl cellulose distends the bowel lumen, giving the bowel a translucency that affords a clear view of the mucosa. Entire small bowel is filled at once. Spot radiographs and overhead radiographs are taken.[14] Laparoscopy is an interesting means for both diagnosis and treatment of MD.[15] However, in none of our cases it was performed.

In some situations like peritonitis due to appendicitis, bowel perforations except MD’s complications, it is better to postpone diverticulectomy. Resection and anastomosis of MD in a purulent abdomen must be avoided if it is not indispensable.[8] In those cases, treatment of MD is delayed for three or four months. Systematic research of MD during all laparotomies includes real risk of dissemination of infection and postoperative adhesions.[3]

Robin *et al*.[16] had a thorough literature search, and they suggested that morbidity rates after resection of incidentally found MD are much lower than those after resection of symptomatic MD. They had suggested the risk score based on four risk factors: male sex, patients younger than 45 years, diverticulae longer than 2 cm and the presence of fibrous band. Resection of MD was suggested by them with a risk score of more than or equal to 6 points. Bani-Hani and Shatnawi[17] compared incidental and symptomatic cases of MD and suggested that resection of incidentally found MD is not associated with increased operative morbidity or mortality.

In our study, during the operation we detected 15 (1.13%) patients with MD. In none of these patients, presence of MD was detected preoperatively. Seven (46.7%) cases were symptomatic due to MD, and the remaining 8 (53.3%) were asymptomatic. Among these, in 2 (13.3%) cases there was heterotopic epithelium. All these patients recovered without any major postoperative complication. We had 28.6% morbidity in symptomatic patients and 12.5% morbidity in asymptomatic patients following resection of MD. In asymptomatic group, only 1 patient had subacute intestinal obstruction treated conservatively. There was no mortality in our study. When compared to complications arising from untreated MD, these complications were negligible. Hence we strongly recommend resection of MD in asymptomatic patients.

## CONCLUSION

In every case of laparotomy for acute abdomen, MD should be searched for, and when present, it should be removed even if asymptomatic because the problems arising as secondary complications are undoubtedly more severe compared to any discomfort following its surgical removal. Preoperative diagnosis is difficult. Technitium-99 m scanning and small bowel enema would be the most accurate investigations. Thorough peritoneal toileting and wound toileting and appropriate higher antibiotics will prevent both intra-abdominal and wound complications.

We recommend that a search for MD in every case of appendicectomy should be conducted, irrespective of whether the appendix is inflamed or not; and if found, diverticulectomy or resection should be performed to avoid complications arising from it.
